# Therapeutic potential of aripiprazole in the treatment of postpartum psychopathological conditions - clinical and hormonal aspects

**DOI:** 10.1192/j.eurpsy.2026.11508

**Published:** 2026-07-31

**Authors:** F. Pepic

**Affiliations:** Clinic for psychiatry, Clinical Center of the University of Sarajevo, Sarajevo, Bosnia and Herzegovina

## Abstract

**Introduction:**

The research was conducted on a sample of 12 patients in the postpartum period, of whom 8 had postpartum depression, and 4 had postpartum psychosis. The influence of aripiprazole on prolactin level and therapeutic outcome was analyzed. In patients with postpartum depression, those who received aripiprazole had a more pronounced decrease in prolactin and a shorter hospitalization. Similar findings were recorded in patients with psychosis. The findings indicate a potential benefit of aripiprazole in the treatment of postpartum disorders.

**Objectives:**

The connection between hormonal changes and psychological disorders in the postpartum period is complex and is the subject of numerous studies. Prolactin, a hormone that increases after childbirth, can be associated with the development of depressive and psychotic symptoms. The aim of this study was to examine the association of prolactin level with the intensity of symptoms and the effect of aripiprazole.

**Methods:**

The sample included 12 patients hospitalized for postpartum psychological disorders. Eight patients were diagnosed with postpartum depression, and four with postpartum psychosis. In patients with postpartum depression, two groups were formed: group A (n=4) received an antidepressant and aripiprazole (5 mg), while group B (n=4) received only antidepressants. For postpartum psychosis, two patients received aripiprazole in addition to olanzapine, and two only olanzapine. Initial and control measurement of prolactin level was performed in both groups.

**Results:**

In patients with postpartum depression, the maximum prolactin level was 785 mIU/L. In Group A there was a 38% drop in prolactin, while in Group B the drop was 17%. The average duration of hospitalization was 18 days for Group A and 26 days for Group B.

In patients with postpartum psychosis, the maximum prolactin level was 1478 mIU/L. A greater therapeutic response and decrease in prolactin was observed in patients who received aripiprazole compared to those who received olanzapine.

During hospitalization, a control measurement of prolactin was carried out, which confirmed a more pronounced drop in the groups that received aripiprazole.

**Conclusions:**

The control measurement of prolactin showed a more pronounced reduction in the group of patients receiving aripiprazole. At the same time, a shorter stay in the hospital and a better therapeutic response were recorded. These findings support the hypothesis that aripiprazole, as a partial agonist of the D2 receptor, can have a beneficial effect in the regulation of postpartum mental disorders along with hormonal stabilization.

Aripiprazole in low doses can have a beneficial therapeutic effect not only on symptoms but also on prolactin regulation. Research limitations are:

- A small sample of respondents
- No randomization

**Disclosure of Interest:**

None Declared

